# Foreign Dentists in England

**Published:** 1889-10

**Authors:** 


### Editorial, Etc.

---

Foreign Dentists in England.—The following from the London *Chemist and Druggist* contains more truth than fiction: "The attempt made in England to boycott foreigners and colonists holding medical and dental degrees has been of late years of so glaring and offensive a nature that foreigners are beginning to retaliate, and in time will ignore all English qualifications, as witness Canada, France, Switzerland, Austria and some American States.

Many of the American dentists, so called in England, are declamatory at times, not to say obtrusive, but the real representative dentist of the United States prefers as a rule to stop at home, where he flourishes."

